# The Importance of Biological Maturation and Years of Practice in Kayaking Performance

**DOI:** 10.3390/ijerph18168322

**Published:** 2021-08-06

**Authors:** Rui António Fernandes, Daniel López-Plaza, Lorena Correas-Gómez, Beatriz Branquinho Gomes, Fernando Alacid

**Affiliations:** 1 Faculty of Sport Sciences and Physical Education, University of Coimbra, 3040-248 Coimbra, Portugal; rui.fernandes@uc.pt (R.A.F.); beatrizgomes@fcdef.uc.pt (B.B.G.); 2 Sport Medicine Chair, Catholic University of Murcia, 30107 Murcia, Spain; dlplaza@ucam.edu; 3 Faculty of Education Sciences, University of Málaga, Andalucia Tech, 29071 Málaga, Spain; lcg@uma.es; 4 Faculty of Education Sciences, Health Research Center, University of Almeria, 04120 Almeria, Spain

**Keywords:** kayaking, years of practice, maturity, performance, anthropometry

## Abstract

Previous canoe sprint studies evaluated the best paddlers of their categories. This investigation aimed to identify the importance of biological maturation and athletes’ experience in kayaking performance and observe possible differences regarding anthropometry, years of practice, and performance. Eighty under 14 years of age (U14) and fifty under 16 years of age (U16) kayakers aged 13.40 ± 0.54 and 15.25 ± 0.61 years were evaluated. Kayakers were assessed for anthropometry (body mass (kg); stretch stature (cm); and sitting height (cm)), performance (time at 3000 m for U14 and 5000 m for U16 kayakers), and somatic maturation (predicted adult height (PAH) and maturity offset). In the U14 kayakers, years of practice, sitting height, and maturity offset showed significant differences (*p* < 0.05) between the Top10 and Middle, and Middle and Bottom10 performance times. Significantly higher (*p* < 0.05) sitting heights were identified between the Top10 and Middle U16 kayakers. Significant differences (*p* < 0.05) were observed for maturity offset and PAH% between the Top10 and Middle groups compared to the Bottom10 group. In conclusion, this research shows differences in the maturity status of young U14 and U16 kayakers, identifying that the more biologically mature individuals, with more years of specific practice, achieved better performances.

## 1. Introduction

The International Canoe Federation recognizes various disciplines, with canoe sprint being, probably, the most popular [1]. In all competitive categories from under 14 and all levels of competition, national or international, the official events are 200 m, 500 m, and 1000 m that are performed in venues duly marked by buoys. Additionally, in sprint canoeing, long-distance competitions of 3000 m for under 14 kayakers and 5000 m for under 16 paddlers and above are held on a predefined circuit that paddlers typically must repeat clockwise. Generally, these competitions have substantial participation of young kayakers, and in many countries, such as in Portugal and Spain, a pre-qualification competition must be organized to ultimately select the paddlers that will participate in the National Championship. Therefore, during these age group competitions and considering the age, participants with different levels of competition experience can be found.

Chronological age, which is calculated as a single time point away from the date of birth, has traditionally been used in sports to group age-grade participants, identify talented performers, and set limits for exercise prescription [2]. Despite this, the literature has demonstrated that individuals of the same chronological age can differ concerning biological maturity [3].

Talent identification programs are designed to identify young athletes with the potential to succeed in senior elite categories [4]. The literature shows that traditional talent identification and development models exclude many talented children from supporting programs [5]. Most of these children seem to beat the early stages of their biological maturation compared to other athletes of the same chronological age or category (late maturers) [6]. Moreover, evidence shows that early specialization is not the only way to achieve expertise [7]. The age of peak competitive performance of elite athletes ranges widely between different sports [8]. Older athletes are found mainly in sports requiring higher tactical skills and nautical sports [9]. In kayaking, the winners of the men’s K1 1000 m event at the Olympic Games of 1996, 2000, 2004, 2008, 2012, and 2016 were, on average, roughly 29 years old. Thus, talent identification and development models must be sensitive to differentiate between an athlete’s adolescent performance level and potential for progression [6]. Previous models showed that talent is usually assessed exclusively by exceptional performances [10], not considering other factors such as maturity status or sport-specific experience. Currently, it seems clear that the performance profiles are multidimensional, and several characteristics should be considered for this purpose [11]. Any talent identification process must acknowledge and account for maturity-related variation in performance [2], avoiding less mature children dropping out of a sport because of a lack of perceived competence or lack of success [12].

From conception to physical maturation in the first twenty years of life, the dominant processes are growth, maturation, and development [13]. Growth is related to the increase in the size of the body as a whole and its parts. Maturation is a process, and maturity is a state. Maturation is related to progress towards biological maturity. It involves changes in body size, body proportion, and composition [3], and these changes may affect performance. Maturation seems to play a significant role in motor skill development, strength, and power. Previous research indicates that childhood is an ideal and opportunistic period to maximize motor skill proficiency [14]. Moran et al. [15], in a meta-analysis of maturation-related variation in adolescent boy athletes, showed that resistance training is more effective during and after peak height velocity in boy athletes. In addition, the interest in this topic in the context of youth sports is evident. Several studies about bio-banding in football (bio-banding is the process of grouping practitioners based on attributes associated with growth and maturation and not chronological age) have been conducted [5,16,17,18]. Previous canoeing studies have focused on analyzing athletes from a morphological and a maturity standpoint [19,20,21,22], considering performance [23]. These studies have evaluated the best paddlers of their categories and reported that the most biologically mature paddlers also showed the best performances.

This approach does not allow access to the entire population of under 14 and 16 kayakers, rendering the design of a more comprehensive and complete evaluation battery impossible. Furthermore, considering the evidence that an individual’s biological maturation influences the talent detection process, it is vital to analyze more heterogeneous groups of kayakers to design these batteries with the sensitivity to evaluate and value the athletic potential of kayakers and not just the immediate performance.

It is also essential to understand the influence that years of specific practice may have on the performance of athletes in younger categories. It has been identified that intense training extended for a minimum of ten years leads to expert performance [24]. Ward et al. [25] showed that, although they start playing soccer at a similar age, elite players began training in a team environment earlier than sub-elite players. This team practice contributes to the difference in performance. However, Olympic world-class athletes started training, competing, and participating in international competitions later than their peers performing at a national level and also competed in other sports, not only their main sport [4]. Another claim for late specialization is that before focusing on a single sport, engagement in multiple sports has benefits in core motor skills and coordination [26]. Cotê et al. [27] stated that the senior international level is attainable with less than five years of practice in the sport. Macnamara et al. [28] reported no difference in the starting age between higher and lesser skilled athletes and also stated that, on average, deliberate practice accounted for 18% of the variance in sports performance. The remaining 82% of the variance may, hypothetically, be explained by other factors. Côté et al. [29] concluded that sports sampling does not harm future expert performance.

Thus, it was hypothesized that biological maturation and years of kayaking practice might influence performance times. Therefore, this investigation aimed to identify the importance of biological maturation and athletes’ experience in kayaking performance and observe possible differences regarding anthropometry, years of practice, and performance times. 

## 2. Materials and Methods

### 2.1. Participants

This study involved 130 young Spanish and Portuguese male kayakers, 80 under 14 years of age (U14) and 50 under 16 years of age (U16), aged 13.40 ± 0.54 and 15.24 ± 0.61 years, respectively, representing 55.2% of the U14 and 34.5% of the U16 kayakers who participated in the championships. Data collection took place at the Spanish and Portuguese National Championships, with about a month of difference. In both competitions, good weather with no wind and racecourse conditions with flatwater and no current were verified. These competitions were organized by the Royal Spanish Canoeing Federation and the Portuguese Canoe Federation.

Athletes were assessed throughout the day, considering their competition schedule. All participants with less than one year of practice were excluded from the assessment, and all had passed the mandatory medical exams necessary to participate in national competitions in both countries. The ethical committee approved the experimental procedures, and before the beginning of the study, written parental or guardian informed consent was obtained.

### 2.2. Anthropometry

All measurements were taken following the procedures described by the International Society for the Advancement of Kinanthropometry by two certified level 3 anthropometrists. Body mass (kg) was determined using a SECA 878 (Digital scale, SECA, Germany); stretch stature (cm) and sitting height (cm) were determined with a SECA 206 (Portable stadiometer, SECA, Germany). Instruments were calibrated before the beginning of each testing session to avoid measurement errors. Relative technical error of measurement [30] was 0.11% for stretch stature, 0.11% for sitting height, and 0.03% for body mass.

### 2.3. Performance

For performance, the official time that each athlete needed to perform the total distance of the race, 3000 m in the case of the U14 and 5000 m for the U16 kayakers, was considered. For that purpose, the results registered by the officials of both competitions were used. All participants were then distributed into groups depending on the years of practice at the time of the assessment: <3 years (Bottom-Exp), ≥3 to <5 years (Mid-Exp), and ≥5 years (Top-Exp); and depending on the performance time: Top10, Middle, and Bottom10.

### 2.4. Maturity Status

Different non-invasive indicators enable understanding the tempo and timing of the biological processes that occur towards the mature state. Somatic maturation was used to evaluate biological development [3], and maturity status was defined by the percentage of the predicted adult height (PAH%) and by the athlete predicted adult height (PAH). The procedure used to estimate adult height was proposed by Khamis and Roche [31]. This method uses the current height, body mass, and mean parental height, which is corrected according to Epstein et al.’s [32] equation. This method assumes that an individual who is close to their mature height is “advanced”, while an individual who is below the predicted adult height for their age is “delayed” [33].

All participants were distributed into groups depending on their maturity status [16] at the time of the assessment: <88% (Bottom%PAH), ≥88% to <92% (Middle%PAH), and ≥92% (Top%PAH) for the U14 category, and <95% (Bottom%PAH), ≥95% to <97% (Middle%PAH), and ≥97% (Top%PAH) for the U16 kayakers. Additionally, maturity offset [34] was used. This method uses chronological age, height, body weight, lower limb length, and sitting height to estimate the distance in years that the subject is from the age at peak height velocity (APHV). The value can be negative if APHV is not yet reached or positive if APHV is surpassed. 

### 2.5. Statistical Analyses

The hypotheses of normality were verified using the Kolmogorov–Smirnov and Shapiro–Wilk tests, while the homogeneity of variance was analyzed using Levene’s test. The comparisons of the group mean for maturation, years of practice, and performance time were performed using a one-way analysis of variance (ANOVA) test when statistical tests revealed no violations of the assumptions of normality and homogeneity. If one-way ANOVA analysis demonstrated significant differences, post hoc Bonferroni tests were conducted to allocate the differences between groups. The Kruskal–Wallis test was used when the normality supposition of the data was not verified, and post hoc tests with Bonferroni corrections were applied. The level of significance was set as *p* < 0.05. Pearson’s correlation coefficient (*r*) was used to determine the relationships between performance, biological maturation, years of practice, and anthropometry. Spearman’s correlation coefficients (*rs*) were calculated when the assumptions of normality were violated. They were considered a very high correlation when 0.9–1, a high correlation when 0.7–0.9, a moderate correlation when 0.5–0.7, a low correlation when 0.3–0.5, and a negligible correlation when 0–0.3 [35]. Statistical analysis was performed using SPSS Statistics 27.0 (SPSS Inc., Chicago, IL, USA).

## 3. Results

The chronologial age, years of practice, anthropometry, maturity, and performance parameters for the U14 and U16 paddlers are presented in Table 1.

### 3.1. Kayakers Grouped by Bands Based on the Percentage of Predicted Adult Height, by Years of Practice, and by Performance

Table 2 summarizes the data related to chronological age, years of practice, anthropometry, maturity, and performance parameters by groups based on PAH% (Bottom%PAH, Mid%PAH, and Top%PAH), years of practice (Bottom-Exp, Mid-Exp, and Top-Exp), and performance (Top10, Middle, and Bottom10) in the U14 category.

Chronological age presented significant differences between the groups based on PAH% (*p* < 0.05) for the three defined maturity groups, with the kayakers from the Top%PAH group being older than the rest (Mid%PAH and Bottom%PAH group). Kayakers from the Top%PAH group were substantially heavier than the rest, particularly the Bottom%PAH group. However, there were no significant differences for the performance variables under study between the three maturity groups in this category, although the best performances were obtained by the Top%PAH (857.47 ± 141.10 s).

When grouped by performance, no significant differences for chronological age were identified. However, it was possible to verify that the Top10 kayakers were significantly more experienced than the Middle and Bottom10 kayakers (4.40 ± 1.26, 3.25 ± 1.48, and 2.60 ± 0.96 years, respectively, *p* < 0.05). Significant differences (*p* < 0.05) were also found between stretch stature (169.55 ± 3.93 cm) and APHV (161.08 ± 8.56 cm) between the Top10 and Bottom10 groups, and PAH% revealed significant differences between the Top10 (92.33 ± 1.16 %, *p* < 0.05) and Middle performance (89.52 ± 3.00 %, *p* < 0.05) time groups. 

As for the U16 kayakers (Table 3), when compared by groups based on PAH%, kayakers from the Top%PAH group (15.75 ± 0.37 years, *p* < 0.05) were significantly older than kayakers from the Mid%PAH (15.29 ± 0.52 years, *p* < 0.05) and Bottom%PAH groups (12.82 ± 0.48 years, *p* < 0.05). When grouped by years of practice, chronological age showed significant differences (*p* < 0.05) between the Bottom-Exp (1.73 ± 0.48 years) and Mid-Exp (3.41 ± 0.49 years) groups, and between the Bottom-Exp (1.73 ± 0.48 years) and Top-Exp groups (6.42 ± 1.10 years). Performance showed significantly better performances (*p* < 0.05) between the Top10 and Bottom10 performance times. At the same time, the comparison by years of practice showed that the kayakers from the Top-Exp group were significantly faster (*p* < 0.05) than the rest of the groups.

In the comparison between groups of performance, kayakers who obtained the Bottom10 performance times revealed a significantly lower chronological age (*p* < 0.05) compared with the remaining groups (Top10 and Middle).

U16 kayakers had significantly (*p* < 0.05) larger body dimensions for sitting height, between Top10 and Middle performances. Additionally, regarding maturity offset and PAH%, the Top10 and Middle groups, when compared to the Bottom10 performances, were significantly (*p* < 0.05) more mature.

### 3.2. Relationship between Performance and Chronological Age, Years of Practice, Anthropometry, and Maturity

Table 4 shows an overview of the relationships between performance time and chronological age, years of practice, anthropometry, maturity, and average velocity for the U14 and U16 groups. For both age groups, negative and significant relationships were found in the years of practice, sitting height, maturity offset, and average velocity. A negligible and moderate correlation was identified in the U14 and U16 groups, respectively, in the analysis of the years of practice (*p* < 0.05, *p* < 0.01, respectively). Interestingly, chronological age showed a significant and negative association with the performance time for the group of U16 kayakers. It is noteworthy that a negative correlation with the time of performance in the competition means better results for kayakers in the respective variables under analysis, with the exception of APHV.

## 4. Discussion

The objective of this study was to analyze the importance of biological maturation and athletes’ experience in specific kayaking performance. As it was hypothesized, biological maturation and years of practice have some influence on performance times.

Building an athlete to reach their maximum possible performance is a long process with many factors that may influence and determine a kayaker’s overall performance. Some of the most common factors affecting performance are technique, experience, maturity status, physiological and anthropometric characteristics, equipment, personality, health, tactics and strategies, nutrition, and environmental conditions [36].

The main findings of this work are that for performance groups of U14 kayakers, regarding years of specific practice, the Top10 athletes showed significantly more years of practice than the Middle and Bottom10 groups. As no significant differences for chronological age were found between these same groups, this suggests that there were athletes who start their specific practice at very young ages compared to their peers in the same category. Concerning performance groups and biological maturation, the Top10 U14 kayakers showed significant differences in maturity offset from the rest of the kayakers (Middle and Bottom10), and significant differences in PAH% only from the Middle kayakers group. Mirwald et al. [34] showed that performance changes are particularly evident just before and during the onset of the peak height velocity. López-Plaza et al. [23] stated significant differences between maturity groups and performance, suggesting that maturity status is a predictor of race performance and may reveal the importance of maturity status at a similar chronological age.

In contrast, for the U16 group, the Top10 kayakers only revealed differences from the Bottom10 athletes for years of practice and chronological age. In the U16 kayakers, both maturity offset and PAH% showed significant differences between the Top10 and the Bottom10 groups. These differences may be casual in both situations, as the Top10 kayakers are just over one year older than the Bottom10 athletes.

According to Ackland et al. [37], basic anthropometric variables have been considered relevant when identifying elite Olympic paddlers. These same characteristics also prove to be important in identifying elite young paddlers [13,15], and additionally, Hamano et al. [31] reported height as a determinant factor in kayaking. However, the results of the U14 group in stretch stature, sitting height, and body mass were below those reported in previous studies with young kayakers [21], probably because previous studies [21,22] assessed the best athletes in their categories, instead of different levels.

Similarly, prior research has reported taller and heavier kayakers [21,22] than the U16 paddlers of the present study. In addition, they were advanced in terms of maturity offset (1.97 ± 0.89, 1.61 ± 0.65 years, respectively). However, focusing on the Top10 kayakers at 5000 m, these were advanced in maturity offset. The results for stretch stature, sitting height, and body mass are similar to those reported in 2019 by López-Plaza et al. [22].

Biological maturation is related to physical performance during adolescence and is more pronounced when comparing boys with a wide range of maturity statuses. Since, generally, athletes of different competitive levels are characterized by an average or advanced maturity status [38], maturation is discriminatory in sports where the best performances are dependent on the physical level [6]. López-Plaza et al. [23] reported that in kayaking and canoeing, the more biologically mature paddlers obtained the best paddling times. The differences in growth and maturation seem to contribute to the selection process in individual sports such as tennis and table tennis [32,33] and team sports such as basketball [39].

In the present study, significant differences between 3000 m performances were observed in the U14 groups only when the kayakers were compared by groups of performance. Despite this observation, there were no significant differences for chronological age. It was observed that the best kayaking times were obtained by more mature kayakers with more years of specific practice, which can contribute to a higher level of technical execution, and this may result from a likely increase in strength. When evaluated by groups based on PAH%, despite significant differences regarding maturity offset and PAH%, differences were not observed in the years of practice. Conversely, when evaluated by groups of experience, differences were observed in years of practice, but not in terms of maturity offset and PAH%, revealing that to verify significant differences in performance, a combination of years of practice and indicators of somatic maturation is necessary for the U14 group.

Previous studies have shown that a larger body size was associated with better performances [40,41], as well as demonstrating chronological age and maturity status as the best predictors of paddling times [23]. This is particularly noticeable in the U16 kayakers, where, in any of the three forms of grouping (groups by bands based on PAH%, by years of practice, and by performance), there were significant differences in chronological age, years of practice, sitting height, maturity offset, and PAH%, with apparently evident repercussions on performance times. However, in both categories, U14 and U16, when compared by performance groups, the best performances were also associated with advanced maturational profiles, maturity offset, and PAH%.

The data obtained in the present research also suggest that more years of specific practice result in better performances. Alves and Silva [42] showed that the Portuguese men’s kayaking team (19.60 ± 1.90 years), in preparation for the Beijing Olympic Games, had 7.3 ± 2.1 years of practice, training of 11.6 ± 0.7 sessions per week, and a mean of 3.2 ± 0.4 h of daily training. Allen and Hopkins [8] stated that differences in the attributes required for success in different sporting events probably contribute to elite athletes’ wide range of peak performance ages.

Regarding the relation with performance, chronological age (*r* = −0.426) and PAH% (*r_s_* = −0.426) only correlated significantly (*p* < 0.01) for the U16 group. Additionally, for the U14 group, performance only correlated significantly with APHV (*r* = −0.426, *p* < 0.05). Moreover, in both the U14 and U16 groups, there were significant correlations between performance and years of practice (*r_s_* = −0.240, *p* < 0.05 and *r_s_* = −0.375, *p* < 0.01, respectively), sitting height (*r_s_* = −0.314 and *r* = −0.375, *p* < 0.05, respectively), and maturity offset (*r_s_* = −0.261, *p* < 0.01 and *r* = −0.493, *p* < 0.05, respectively). López-plaza et al. [23] found that chronological age (*r* = −0.720, −0.600, and −0.712), height (*r* = −0.495, −0.433, and −0.510), sitting height (*r* = −0.514, −0.622, and −0.643), body mass (*r* = −0.441, −0.325, and −0.423), and maturity offset (*r* = −0.628, −0.674, and −0.731) were all significantly (*p* < 0.01) correlated with performance at 1000, 500, and 200 m, respectively. Forbes et al. [43] also reported that age (*r* = −0.59), height (*r* = −0.81), and sitting height (*r* = −0.85) were all significantly (*p* < 0.05) correlated with 1000 m performance. Regarding sitting height, it should be noted that coaches and athletes may try to alter the boat setup in order to counteract a possible lower sitting height, namely, by raising the boat seat height of the kayaker. However, this can influence the mechanical efficiency of the paddling technique [44] and probably demand a longer paddle, which, initially, can lead to a reduction in boat speed [45].

The distances analyzed in this investigation were different in comparison with previous studies. This fact cannot be ignored since the requirements and specificities of the effort are different. Additionally, the 1000 m trials are typically carried out in a straight line, whereas the long distance trials such as the 3000 and 5000 m trials are performed in a circuit, which may imply a more significant influence of chance on performance. In circuit events, athletes can navigate in groups on watercourses with a variable width and depth and with the need to go around the buoys several times, thus increasing the probability of the occurrence of a misfortune such as the vessel capsizing, failing the number of laps, shortcutting or increasing the route distance, or breaking the rudder. Additionally, the present study has limitations, such as the impossibility of assessing all competition participants. In addition, the specific nature of the competition, ability to ride the opponent’s wave, tactical decision making, problems due to equipment malfunction (paddle and kayak), and the fact that there was a qualifying event on the morning of the competition may all have negatively influenced performance.

## 5. Conclusions

The involvement of children in competitive sports today is a widespread and multifaceted reality. Thereby, young athletes are subjected to considerable changes that determine different effects on training and performance. There are two ways in which young athletes can improve their performance: training and growth.

This research has shown differences in the maturity status of young U14 and U16 kayakers, identifying that the more biologically mature individuals were also those who had larger bodies and the best kayaking performances. Additionally, the kayakers with more years of specific practice were the ones that achieved better performances.

Despite focusing on populations that compete in Spain and Portugal, this study is unique because it attempts to assess all the participating kayakers in the competition and evaluate competitions held in circuits that carry distinct particularities. In contrast, previous studies considered only the best in their categories in straight-line competitions. Kayaking is a sport where technical ability seems to be critical, and technical skills may influence performance. Future studies should consider the possibility of evaluating the technical skills of the athletes and assessing larger samples. It would also be interesting if future investigations focused on the relative age effect in kayaking and tactical decision making in circuit competitions. To conclude, it is essential to focus on young athletes’ potential to develop towards expert performance. The present study’s findings underline the relevance of creating evaluation batteries that contain information on maturity status and years of specific practice. 

## Figures and Tables

**Table 1:** Mean values (± SD) of chronological age (CA), years of practice, anthropometry, maturity, and performance parameters in U14 and U16 paddlers.

	Mean (± SD)	
	U14 (*n* = 80)	U16 (*n* = 50)
CA (years)	13.40 ± 0.54	15.24 ± 0.61
Years of Practice (years)	3.29 ± 1.46	4.12 ± 2.01
Anthropometry		
Stretch Stature (cm)	163.75 ± 9.22	172.52 ± 5.59
Sitting Height (cm)	85.49 ± 4.98	90.75 ± 3.50
Body Mass (kg)	55.06 ± 10.73	63.68 ± 6.78
Maturity		
Maturity Offset (years)	−0.06 ± 0.78	1.61 ± 0.65
APHV (years)	13.46 ± 0.64	13.62 ± 0.52
PAH (cm)	182.07 ± 6.61	179.77 ± 5.44
PAH (%)	89.90 ± 3.01	96.06 ± 1.73
Performance		
3000 m time (s)	870.32 ± 116.14	-
5000 m time (s)	-	1450.95 ± 90.52
Average Velocity (m.s^−1^)	3.50 ± 0.44	3.45 ± 0.21

CA: chronological age; APHV: age at peak height velocity; PAH: predicted adult height.

**Table 2:** Mean (± SD) for chronological age (CA), years of practice, anthropometry, maturity, and performance parameters for bands based on the percentage of predicted adult height (Bottom%PAH, Mid%PAH, and Top%PAH), grouped by years of practice (Bottom-Exp, Mid-Exp, and Top-Exp), and grouped considering performance (Top10, Middle, and Bottom10) for under 14 kayakers.

	Under 14 (*n* = 80) Mean (± SD)								
	Groups by Bands Based on PAH%			Groups by Years of Practice			Groups by Performance		
	Bottom%PAH (*n* = 19)	Mid%PAH (*n* = 37)	Top%PAH (*n* = 24)	Bottom-Exp (*n* = 29)	Mid-Exp (*n* = 36)	Top-Exp (*n* = 15)	Bottom10 (*n* = 10)	Middle (*n* = 60)	Top10 (*n* = 10)
CA (years)	12.82 ± 0.48	13.44 ± 0.57 *	13.78 ± 0.31 ^†§^	13.38 ± 0.52	13.40 ± 0.56	13.43 ± 0.52	13.64 ± 0.62	13.33 ± 0.51	13.52 ± 0.53
Years of Practice (years)	3.39 ± 1.63	3.16 ± 1.39	3.41 ± 1.47	1.82 ± 0.48	3.48 ± 0.52 *	5.66 ± 0.61 ^†§^	2.60 ± 0.96	3.25 ± 1.48	4.40 ± 1.26 ^†§^
Anthropometry									
Stretch Stature (cm)	152.42 ± 8.24	165.31 ± 5.59 *	170.32 ± 5.90 ^†^	162.79 ± 9.92	165.67 ± 8.57	161.00 ± 8.91	161.08 ± 8.56	163.23 ± 9.60	169.55 ± 3.93 ^†^
Sitting Height (cm)	80.21 ± 4.63	85.98 ± 3.75 *	88.90 ± 3.32 ^†§^	84.83 ± 5.18	86.65 ± 4.85	83.96 ± 4.52	82.85 ± 4.19	85.29 ± 5.08	89.33 ± 2.51 ^†§^
Body Mass (kg)	46.87 ± 12.40	54.42 ± 7.72*	62.52 ± 8.19 ^†§^	53.40 ± 11.98	57.33 ± 9.92	52.82 ± 9.55	54.62 ± 12.50	54.16 ± 10.98	60.92 ± 4.41
Maturity									
Maturity Offset (years)	−1.02 ± 0.56	−0.00 ± 0.52 *	0.61 ± 0.39 ^†§^	−0.16 ± 0.82	0.10 ± 0.75	−0.24 ± 0.72	−0.25 ± 0.63	−0.12 ± 0.81	0.51 ± 0.41 ^†§^
APHV (years)	13.85 ± 0.75	13.44 ± 0.57 *	13.17 ± 0.45 ^†^	13.54 ± 0.65	13.29 ± 0.59	13.67 ± 0.63	13.89 ± 0.56	13.46 ± 0.63	13.01 ± 0.41 ^†^
PAH (cm)	178.35 ± 8.02	183.18 ± 5.46 *	183.30 ± 6.17 ^†^	181.40 ± 6.60	183.58 ± 6.77	179.7 ± 5.66	179.50 ± 6.55	182.23 ± 6.75	183.68 ± 6.5
PAH (%)	85.44 ± 1.77	90.24 ± 1.07 *	92.91 ± 0.83 ^†§^	89.67 ± 2.91	90.23 ± 3.06	89.55 ± 3.01	89.73 ± 3.38	89.52 ± 3.00	92.33 ± 1.16 ^§^
Performance									
3000 m time (s)	889.10 ± 99.35	869.01 ± 107.90	857.47 ± 141.10	902.59 ± 111.00	865.66 ± 126.66	819.11 ± 79.53	1093.03 ± 44.13	859.37 ± 70.69 *	713.29 ± 19.34 ^†§^
Average Velocity (m.s^−1^)	3.41 ± 0.37	3.50 ± 0.41	3.58 ± 0.53	3.36 ± 0.38	3.53 ± 0.47	3.69 ± 0.37	2.75 ± 0.11	3.51 ± 0.28 *	4.21 ± 0.11 ^†§^

CA: chronological age; APHV: age at peak height velocity; PAH: predicted adult height. * Significant difference (*p* < 0.05) between Bottom%PAH and Mid%PAH bands, between Bottom-Exp and Mid-Exp, and between Bottom10 and Middle. ^†^ Significant difference (*p* < 0.05) between Bottom%PAH and Top%PAH, between Bottom-Exp and Top-Exp, and between Bottom10 and Top10. ^§^ Significant difference (*p* < 0.05) between Mid%PAH and Top%PAH bands, between Mid-Exp and Top-Exp, and between Middle and Top10.

**Table 3:** Mean (± SD) for chronological age (CA), years of practice, anthropometry, maturity, and performance parameters for bands based on the percentage of predicted adult height (Bottom%PAH, Mid%PAH, and Top%PAH), grouped by years of practice (Bottom-Exp, Mid-Exp, and Top-Exp), and grouped considering performance (Top10, Middle, and Bottom10) for under 16 kayakers.

	Under 16 (*n* = 50) Mean (± SD)								
	Groups by Bands Based on PAH%			Groups by Years of Practice			Groups by Performance		
	Bottom%PAH (*n* = 16)	Mid%PAH (*n* = 18)	Top%PAH (*n* = 16)	Bottom-Exp (*n* = 13)	Mid-Exp (*n* = 18)	Top-Exp (*n* = 19)	Bottom10 (*n* = 10)	Middle (*n* = 30)	Top10 (*n* = 10)
CA (years)	14.66 ± 0.32	15.29 ± 0.52 *	15.75 ± 0.37 ^†§^	14.75 ± 0.39	15.43 ± 0.60 *	15.38 ± 0.56 ^†^	14.62 ± 0.29	15.31 ± 0.58 *	15.65 ± 0.41 ^†^
Years of Practice (years)	2.96 ± 1.70	4.02 ± 1.95	5.37 ± 1.95 ^†^	1.73 ± 0.48	3.41 ± 0.49 *	6.42 ± 1.10 ^†§^	2.35 ± 0.88	4.41 ± 2.23 *	5.00 ± 1.47 ^†^
Anthropometry									
Stretch Stature (cm)	171.60 ± 5.96	170.94 ± 4.40	175.21 ± 5.74	171.12 ± 6.19	172.24 ± 5.28	173.74 ± 5.48	170.04 ± 4.71	172.73 ± 5.56	174.38 ± 6.12
Sitting Height (cm)	89.57 ± 3.70	89.93 ± 2.66	92.84 ± 3.39 ^†§^	88.84 ± 3.21	90.29 ± 2.85	92.48 ± 3.59 ^†^	88.51 ± 2.90	90.98 ± 3.17	92.30 ± 4.14 ^†^
Body Mass (kg)	61.71 ± 7.85	62.52 ± 4.56	66.96 ± 6.91	61.23 ± 7.06	64.28 ± 7.10	64.78 ± 6.18	58.35 ± 5.44	64.79 ± 6.32 *	65.68 ± 7.20 ^†^
Maturity									
Maturity Offset (years)	1.13 ± 0.54	1.52 ± 0.44	2.19 ± 0.50 ^†§^	1.08 ± 0.50	1.66 ± 0.60 *	1.93 ± 0.58 ^†^	0.93 ± 0.45	1.69 ± 0.52 *	2.05 ± 0.69 ^†^
APHV (years)	13.53 ± 0.56	13.76 ± 0.44	13.56 ± 0.55	13.67 ± 0.50	13.77 ± 0.44	13.45 ± 0.57	13.68 ± 0.43	13.61 ± 0.54	13.59 ± 0.58
PAH (cm)	182.48 ± 5.86	178.09 ± 4.86	178.94 ± 4.85	180.89 ± 6.68	178.64 ± 4.46	180.06 ± 5.45	180.12 ± 4.96	176.65 ± 5.89	179.75 ± 4.93
PAH (%)	90.03 ± 0.91	96.24 ± 0.67 *	97.90 ± 0.67 ^†§^	94.61 ± 1.54	96.41 ± 1.69 *	96.73 ± 1.34 ^†^	94.41 ± 1.67	96.15 ± 1.51 *	97.45 ± 1.02 ^†^
Performance									
5000 m time (s)	1489.59 ± 83.36	1450.55 ± 101.23	1412.77 ± 71.07 ^†^	1532.66 ± 64.16	1439.57 ± 89.53 *	1405.83 ± 69.90 ^†^	1583.36 ± 30.49	1445.01 ± 53.29 *	1336.39 ± 18.60 ^†§^
Average Velocity (m.s^−1^)	3.36 ± 0.18	3.46 ± 0.23	3.54 ± 0.17 ^†^	3.26 ± 0.13	3.48 ± 0.20 *	3.56 ± 0.17 ^†^	3.16 ± 0.06	3.46 ± 0.12 *	3.74 ± 0.05 ^†§^

CA: chronological age; APHV: age at peak height velocity; PAH: predicted adult height. * Significant difference (*p* < 0.05) between Bottom%PAH and Mid%PAH bands, between Bottom-Exp and Mid-Exp, and between Bottom10 and Middle. ^†^ Significant difference (*p* < 0.05) between Bottom%PAH and Top%PAH, between Bottom-Exp and Top-Exp, and between Bottom10 and Top10. ^§^ Significant difference (*p* < 0.05) between Mid%PAH and Top%PAH bands, between Mid-Exp and Top-Exp, and between Middle and Top10.

**Table 4:** Correlations between chronological age, years of practice, anthropometry, and maturity parameters with performance in U14 and U16 kayaker groups (*r* and *r_s_*).

	Under 14 (*n* = 80)	Under 16 (*n* = 50)
	3000 m	5000 m
Chronological Age	0.030	−0.426 **^+^
Years of Practice	−0.240 *ᶺ	−0.535 **ᶺ
Anthropometry		
Stretch Stature	−0.278 *ᶺ	−0.223
Sitting Height	−0.314 **ᶺ	−0.375 **^+^
Body Mass	−0.141	−0.168
Maturity		
Maturity Offset	−0.261 *ᶺ	−0.493 **^+^
APHV	0.305 **^+^	0.112
PAH	−0.215	−0.020
PAH (%)	−0.202	−0.393 **ᶺ
Performance		
Average Velocity	−0.988 **^+^	−0.998 **^+^

APHV: age at peak height velocity; PAH: predicted adult height. * Significant differences (*p* < 0.05). ** Significant differences (*p* < 0.01). ^+^ Pearson’s and ᶺ Spearman’s correlations.

## Data Availability

The data presented in this study are available on request from the authors.
